# What do general practitioners know about ADHD? Attitudes and knowledge among first-contact gatekeepers: systematic narrative review

**DOI:** 10.1186/s12875-016-0516-x

**Published:** 2016-09-07

**Authors:** Mimi Tatlow-Golden, Lucia Prihodova, Blanaid Gavin, Walter Cullen, Fiona McNicholas

**Affiliations:** 1 Department of Child and Adolescent Psychiatry, School of Medicine and Medical Science, University College Dublin, C323, Health Sciences Building, Belfield, Dublin 4, Ireland; 2 Royal College of Physicians, Dublin, Ireland; 3 Department of General Practice, School of Medicine and Medical Science, University College Dublin, Dublin, Ireland; 4 Lucena Clinic, Rathgar, Dublin, Ireland; 5 Our Lady’s Children’s Hospital, Crumlin, Dublin, Ireland

**Keywords:** Attention-deficit hyperactivity disorder, ADHD, General practice, Family practice, Gatekeepers, Attitudes, Systematic review, Training

## Abstract

**Background:**

Attention Deficit Hyperactivity Disorder (ADHD) is a common childhood disorder with international prevalence estimates of 5 % in childhood, yet significant evidence exists that far fewer children receive ADHD services. In many countries, ADHD is assessed and diagnosed in specialist mental health or neuro-developmental paediatric clinics, to which referral by General (Family) Practitioners (GPs) is required. In such ‘gatekeeper’ settings, where GPs act as a filter to diagnosis and treatment, GPs may either not recognise potential ADHD cases, or may be reluctant to refer. This study systematically reviews the literature regarding GPs’ views of ADHD in such settings.

**Methods:**

A search of nine major databases was conducted, with wide search parameters; 3776 records were initially retrieved. Studies were included if they were from settings where GPs are typically gatekeepers to ADHD services; if they addressed GPs’ ADHD attitudes and knowledge; if methods were clearly described; and if results for GPs were reported separately from those of other health professionals.

**Results:**

Few studies specifically addressed GP attitudes to ADHD. Only 11 papers (10 studies), spanning 2000–2010, met inclusion criteria, predominantly from the UK, Europe and Australia. As studies varied methodologically, findings are reported as a thematic narrative, under the following themes: *Recognition rate; ADHD controversy (medicalisation, stigma, labelling); Causes of ADHD; GPs and ADHD diagnosis; GPs and ADHD treatment; GP ADHD training and sources of information; and Age, sex differences in knowledge and attitudes*.

**Conclusions:**

Across times and settings, GPs practising in first-contact gatekeeper settings had mixed and often unhelpful attitudes regarding the validity of ADHD as a construct, the role of medication and how parenting contributed to presentation. A paucity of training was identified, alongside a reluctance of GPs to become involved in shared care practice. If access to services is to be improved for possible ADHD cases, there needs to be a focused and collaborative approach to training.

**Electronic supplementary material:**

The online version of this article (doi:10.1186/s12875-016-0516-x) contains supplementary material, which is available to authorized users.

## Background

Attention-deficit hyperactivity disorder (ADHD) is a common behavioural disorder of childhood, characterised by over-activity, impulsivity and inattention, and is recognised to cause significant personal, academic and social functioning. Marked variation exists in cited prevalence rates, which range from as high as 26 % [1, 2] to lows of 1 %, but once methodological differences are controlled for, international studies suggest a 5–7 % childhood prevalence [3–5] with an accepted worldwide pooled prevalence of 5.29 %. Up to two-thirds of young people who meet criteria for ADHD receive neither diagnosis nor services, as indicated in large-scale national data sets and community screening. In the UK, the British Child and Adolescent Mental Health Survey found fewer than 1 in 3 young people with ADHD had seen a relevant health specialist [6]. In Ireland, parent reports in a nationally representative sample of 8568 nine-year-olds [7] indicate that only 1.2 % children had been diagnosed with ADHD. Under-diagnosis and under-treatment were also reported in a Netherlands community study of 9 year olds with ADHD [8]. Other studies highlight complex diagnosis rates, with evidence of both over- and under-recognition of ADHD within the same jurisdiction, and prevalence influenced by race, ethnicity, gender, and age [9, 10].

In general, identifying mental health difficulties in children and young people is a challenge in primary care [11]. Reviews and studies in Australia, the UK, the US, Finland, the Netherlands and elsewhere have established that only about a third of children and adolescents with mental health problems receive specialised care in child and adolescent mental health services (CAMHS) [12], although the proportions vary internationally, with up to 40 % children with mental health difficulties in contact with any service in the US, compared to up to 29 % elsewhere [13]. For example, in a UK community sample, 74 % children (5–11 years) who met ‘SDQ caseness’ criteria had not been recognised by their GP as having a mental health difficulty [14]. In the Netherlands, just 14 % of children whose parents or teachers thought they had a mental health problem were recognised and diagnosed in general practice; many were instead identified by school personnel [15]. GP interview techniques, the availability and use of screening measures, level of familiarity with a child, and GP training are all thought to influence the recognition of childhood mental health difficulties [16].

Within a context of generally poor recognition of children’s mental health difficulties in primary care, ADHD may present additional challenges, as it remains a socially contested diagnosis, and the subject of on-going debate in popular media as well as in medical professions. Regarding the appropriateness of psychotropic prescribing for children, both reasoned concerns as well as more critical views are found [10, 17–19]. Sayal proposes that in the US, physician expertise and confidence, and an acceptance of ADHD medication use, contribute to a higher level of ADHD recognition compared to other jurisdictions [13]. Beyond national-level variation, studies also indicate considerable local differences in access and uptake of ADHD treatment [17], highlighting the existence of other important social, medical and attitudinal factors [17, 20].

For example, despite extensive research, the *aetiology* of ADHD and the relationship of neurobiology to symptoms remain poorly understood, leaving the field open to multiple interpretations [20, 21]. Furthermore, the evidence for ADHD *treatment* continues to be actively debated, as evidenced by the recent Cochrane meta-analysis of methylphenidate (MPH) effectiveness in childhood ADHD and the subsequent published debate [22–25]. In stark contrast to previously cited reports of high efficacy of stimulants (reflected in recommendations for use in clinical guidelines) [26, 27], the Cochrane review authors concluded that although MPH improves teacher-rated ADHD symptoms, general behaviour, and parent-rated quality of life, almost all the evidence was of “very low quality” [22]. In many responses critiquing this finding, clinicians, researchers and others robustly defended the evidence for MPH, taking issue with the review methodology, which they argued deviated from typical Cochrane procedure, with an inflation of study bias and excessive downgrading of the quality of evidence [24, 25].

In addition to debates over aetiology and treatment, further features of the medical/social context that may affect ADHD diagnosis, referral and treatment are medical service structures and ADHD treatment guidelines. Regarding *service structures*, in many countries, GPs in primary care are service ‘gatekeepers’: for many conditions, including ADHD, they do not diagnose or initiate treatment but rather refer to relevant specialists. In such settings, patients and their families are precluded or discouraged from consulting specialists directly without GP referral – examples are 19 countries in Europe, Canada, Australia and New Zealand [28–31]. This contrasts with the situation in the US and other countries, where primary care physicians predominantly provide ADHD diagnosis and treatment and may be accessed directly [20]. ‘Gatekeeper’ systems [28] generate multiple stages or ‘filters’ in the help-seeking process: for example, parents’ interpretations of a child’s behaviour and decisions to consult a GP are important [13, 32, 33], but GPs hold the key to the pathway to care [28–33]. This practice is under-researched [34] but, as families require GP referral to access diagnosis or treatment, GP attitudes and knowledge of a condition are crucial.

Recommendations in guidelines, regarding who may diagnose ADHD and initiate treatment, further underscore GPs’ gatekeeper role. Reviews of guidelines [27, 35] note that the American Academy of Pediatricians (AAP) and the American Academy of Child and Adolescent Psychiatrists (AACAP) guidelines (which are applied in other countries outside the US) identify family physicians among the professionals who may diagnose ADHD. In contrast, national ADHD guidelines in the UK, Canada, Scotland, New Zealand and pan-European bodies recommend diagnosis only by specialists in secondary services – specialists to whom GPs control referral.

In sum, GPs in gatekeeper settings are charged with identifying children with suspected ADHD and referring them to specialist secondary services, but they do not engage with diagnosis or initiate treatment. Unlike many other psychiatric diagnoses, the nature of ADHD and its optimal treatment, despite being agreed by most psychiatrists and across the world in national treatment guidelines, is disputed by other professionals and questioned in the media [2, 10, 18, 36]; and in many gatekeeper jurisdictions, the rates of children with ADHD attending services are very low. In this context, this review aimed to examine studies of GPs’ knowledge regarding ADHD, focusing on those contexts where GPs are first-contact gatekeepers to mental health services. The study sought to answer the following questions: How do GPs who operate in a gatekeeper setting self-rate their recognition of ADHD in children and young people? Do they consider ADHD to be a valid diagnosis? What do they identify as causing ADHD? Which treatments do they believe are effective? What role do they believe they should play in the pathway to specialist service referral for ADHD?

## Method

The systematic review was carried out following the Preferred Reporting Items for Systematic Reviews and Meta-Analysis (PRISMA) guidelines [37]. The search strategy was designed to be as extensive as possible to identify all possible eligible studies, which were then refined by applying inclusion and exclusion criteria. The electronic search included the following databases: MEDLINE, EMBASE (Elsevier), CINAHL Plus (EBSCO), Scopus, PsycArticles (Proquest), PsycINFO (Proquest), Social Services Abstracts, Applied Social Sciences Index and Abstracts (ASSIA). Reference lists of included studies were screened to ensure literature saturation.

### Search terms and outcome

The primary search in abstracts included three main terms and their variations: *general* (family), *physician* (doctor, primary care, practitioner) and *attention deficit hyperactivity disorder* (ADHD, attention deficit). The search yielded 3776 studies. Duplicates were removed and two researchers independently reviewed 3023 titles and abstracts according to the inclusion and exclusion criteria listed below.

### Inclusion criteria

1. Studies of GPs’ attitudes/knowledge regarding ADHD in children and/or adolescents
2. If more than one profession is studied, GP findings are reported separately
3. Studies in countries where GPs are first-contact gatekeepers
4. Published in or after 1994, when DSM-IV was published
5. Published prior to January 2015
6. English language
7. Published in peer-reviewed publications

### Exclusion criteria

1. Books, chapters and book reviews; clinician review notes, case studies, clinical practice guidelines or recommendations; opinion pieces and commentaries;
2. Studies of ADHD prevalence; medication adherence; ADHD-related physical health in primary care; adult ADHD; and studies making only passing reference to ADHD;
3. Studies of first-contact paediatricians or child and adolescent psychiatrists, whose training, knowledge and recognition are likely to differ substantially from GPs

**Fig. 1:**
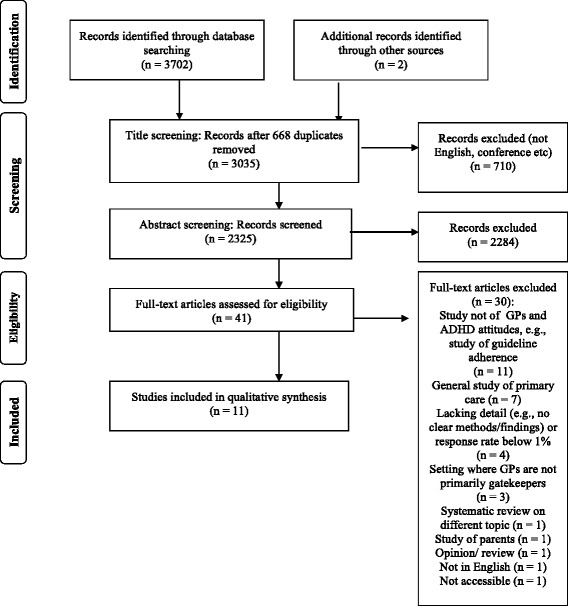
Flow diagram of the systematic review, modified from Moher et al., 2009 [37]

At title screening, 710 studies were excluded and 2284 were excluded after abstract screening. Predominant reasons for exclusion included studies reporting on professions other than GPs, or GP data not being reported on separately (*n* = 1136); studies of ADHD measures or medication efficacy (*n* = 557); or studies where ADHD was included with other general mental health difficulties, but not reported on separately (*n* = 250). At full-text review, 41 studies were examined and 30 were excluded (Fig. 1). Eleven studies did not examine GPs’ ADHD attitudes; most of these were studies of US-based practitioners’ (paediatricians and/or family practitioners) adherence to American Academy of Pediatrics ADHD Guidelines, rather than their attitudes regarding ADHD. Two studies were excluded from countries (South Africa, Singapore) where GPs are not reported to be gatekeepers (i.e., they may treat ADHD themselves). Finally, a recent extensive systematic review of barriers in pathways to care for ADHD [37] was identified, but the studies listed within this were already identified and included by the original search. This review was therefore also excluded leaving 11 papers retained for synthesis (Fig. 1). Table 1 lists all included studies with their key characteristics and findings.

**Table 1:** Studies included in the review of gatekeeper GP attitudes and knowledge regarding ADHD

Authors, location	Aim and design	Participants, measure, analysis	Findings, conclusions	Strengths and limitations
Quantitative studies				
Sayal et al. (2002) [38] UK - London	Hyperactivity in children: compare those who had passed through all service filters, with children in community who had not. Quantitative – identify predictors of GP recognition	Strengths and Difficulties Questionnaire (SDQ) sent to 3218 parents of children 5–11 years in one London area to identify hyperactivity; *n* = 1194 completed (40 % response; SDQ score 6+ *n* = 248, 21 %). 4 groups: No GP attendance; GP attender (referred); GP attender (recognised, not referred); GP attender (unrecognised). Logistic regression; identify predictors of recognition	Only 12 % children with pervasive hyperactivity in community sample were in contact with CAMHS, though 74 % had seen GP in past year. Parent perception of problem the only significantly predictor of GP attendance (hyperactivity, school burden not significant). Non-recognition by GP was main barrier to specialist services. Only comorbid conduct problem or parent referral request predicted GP recognition, after controlling for significant predictor variables. As most high-risk children attend primary care, ADHD could be identified there, but GPs may not recognise it if parent is unaware or reticent.	Strong study design and excellent response rate for a community-based study
Ball (2001) [39] UK - Wales	GPs’ views of ADHD management Quantitative survey	150 GPs (68 % response) Postal survey. GP experience, familiarity with ADHD, methylphenidate. Views of professionals’ roles, prescribing practice, training needs. Descriptive frequency analysis	85 % GPs had a child with ADHD in their practice, 89 % prescribed methylphenidate, 94 % with overview of child psychiatrist/paediatrician. No GP thought GPs should initiate prescribing; 46 % prepared to repeat prescribe; 54 % said primary care could monitor physically; 64 % psychiatry should monitor clinically. 6 % formal ADHD training, 5 % conference/ course, 29 % journal article, 21 % media e.g., television, magazines. 84 % wanted further ADHD training (68 % preferred tutorial or lecture, 27 % written, 5 % phone). GPs overloaded, reluctant to take on more. Study suggests CAMHS need to provide ADHD training ADHD for GPs and engage in discussions re shared care	
Heikkinen et al. (2002) Finland [45]	Primary care health centre GPs’ self-rated child psychiatric skills. Quantitative survey	499 GPs (66 % response) 16-item postal questionnaire (5-point Likert scale) on self-rated ability regarding children’s mood, conduct and other disorders Descriptive frequency analysis	Primary care health centre GPs rated their child psychiatric skills as inadequate in many domains. 41 % rated skill at identifying ADHD in school-aged children as adequate. Medical training including CME appears to focus less on psychiatric than physical problems and GPs may not consider child psychiatry to be clearly within the primary health care remit	Self-report of diagnostic ability. Only 1 item regarding ADHD.
Shaw et al. (2002) [42] Australia - Queensland	Assess GP ADHD knowledge, and actual and potential roles in ADHD management Quantitative survey	399 GPs (76 % response) Randomly selected from RACGP Directory Survey piloted with GPs, parents, health sociologists & statistician. Survey explored demographics & GP ADHD beliefs: existence; causes; diagnosis; practice, management; beliefs re GP role. Diagnostic criteria for ADHD, OCD, CD, anxiety & depression presented (*n* = 16); participants asked to assign each to a diagnosis. Descriptive frequency analysis; internal consistency of relevant factors; chi-square tests compared GP age, gender, rural/urban	*%s represent GP agreement with items* *Aetiology*: Family disruption 97 %; parenting 77 %; poor discipline 75 %; temperament 77 %; brain abnormality 70 %; food 12 %; TV 7 %; video games 5 % birth trauma 4 %; education 3 %. Children with behaviour problems do not have ADHD 76 %; ADHD over-diagnosed 55 %. GPs lack knowledge of child behaviour problems 74 %; ODD/CD symptom mis-identified as ADHD 23–33 %. *1st line treatment*: Behavioural 51 %, stimulants 43 %. Stimulants: 17 % always inappropriate, 86 % can be abused, 40 % addictive. *GPs identified their roles as* provisional diagnosis, referral; monitoring assistance (height, weight, appetite, sleep); psycho-education; school liaison. GPs wanted greater knowledge. School input recommended for diagnosis but not sourced systematically. Most felt assessing children for ADHD best undertaken by specialists within MDT. *Barriers to greater GP involvement:* resources; addiction concern; child behaviour problems complex; lack of ADHD training. GPs diagnosed only 1–5 ADHD cases a year yet saw >550 children 4–16, so under-diagnosis likely. GP confusion about ADHD, mood disorders, disruptive behavioural disorders; weak knowledge of ADHD & comorbidities was weak; low confidence in diagnosing and managing.	Large-scale random sample; very high response rate. Many T/F response options, so chance responding is high Detailed and wide-ranging questionnaire
Miller et al. (2005) [44] Canada – British Columbia	GP self-report of comfort, skill, care of children with behavioural, emotional problems Quantitative survey, inferential analyses	405 GPs (64 % response) stratified by Health Board region. Postal questionnaire; 22 items developed through consultation. Principal Components Analysis (PCA) on comfort/skill items. Repeated measures ANOVAs; multiple linear regressions and logistic regressions	PCA: Comfort, skill not distinct – loaded onto single component for each type of problem GP self-efficacy (comfort/skill ratings) for each problem related to CME as well as to belief that problems are significant and that GPs have a role in them. Possible that CME effects may be due not only to knowledge acquisition but mediated through effects on attitudes and beliefs. Need to bolster GP confidence, alter attitudes, especially re ADHD & behavioural difficulties	Large-scale study with stratified sample and excellent response rate, and inferential analyses.
Salt et al. (2005) [40] UK - London	GP perceptions of ADHD and its management in primary care Mixed methods: Quantitative survey and focus groups (see below)	93 GPs (52 % response) in one London Primary Care Trust Questionnaire: 55 ADHD items: origins (16), symptoms (10), attitudes (9), treatments (9), all dichotomous; shared care (5 items; 7 response options), referral (6 response options). Frequency analysis	*Questionnaire* *ADHD causes* Most cited genetics, chemical imbalance, quality of parenting, family type; peers, environment chemicals, poverty, ethnicity, social class least cited. *Diagnostic criteria*: most included inattention, hyperactivity, impulsivity, but > 75 % cited non-DSM ‘educational underachievement’, ‘antisocial behaviour’ and ‘sleep problems’ as symptoms. *Treatment*: 92 % methylphenidate, followed by family & behaviour therapy. Specialist should be responsible, including monitoring. *Attitudes, knowledge*: ADHD controversial 90 %; media influences attitudes 90 %; patients can be stigmatized, disadvantaged by ADHD diagnosis 79 %; parents invested in child ADHD diagnosis as it shifts blame 44 %. ADHD exists after childhood 85 %	Small local sample, just one primary care trust. Most items had T/F response options, so chance responding is high Detailed questionnaire and associated qualitative section enables deeper interpretation
Ghanizadeh & Zarei (2010) [46] Iran - Shiraz	GP ADHD knowledge Quantitative survey	665 GPs; 74 % response. Postal questionnaire, 20 items (dichotomous response), previously used to assess knowledge among teachers & pharmacists Frequency analyses	*ADHD causes*: 37 % sugar, food additives, 53 % chaotic, dysfunctional family, 90 % parenting, spoiling, 83 % children with ADHD misbehave because they don’t want to obey rules, do assignments; *ADHD nature*: 93 % ADHD is not lifelong; 20 % it is not serious; 75 % can be managed with medication; 71 % psychiatrist should manage; 21 % psychologist should manage; 97 % psychological support needed. *Treatment*: half against methylphenidate use except if severe. *ADHD information*: 10 % passed special courses on ADHD; 32 % info from medical journals, 25 % from media, magazines, 18 % from colleagues	All items had T/F response options, so chance responding is high. Excellent response rate from wide-ranging sample
Qualitative studies				
Klasen & Goodman (2000) [47] & Klasen (2000) [41] UK - London	Examine attitudes of parents and GPs regarding hyperactivity including barriers to treatment Qualitative design	10 GPs from central London, several with academic appointments or interest in children’s services. Also with 29 parents of hyperactive children, selected purposively to achieve range of views. Semi-structured interviews	3 clusters of GP attitudes to ADHD (1) ADHD labels, disempowers active children; reframe ADHD as poor parenting (2) Sceptical, confused by contradictory expert opinions; discourage medicalization, diagnosis is stigmatising; (3) Sceptical, diagnosis can be useful; aware of own limitations; sympathetic attitude to parents. No GP had ever given a diagnosis; believed to be task of specialists. GPs’ decisions about referral were moral as well as medical, based on beliefs that diagnosis can stigmatise, and make children passive and dependent. They often failed to recognize that diagnosis can legitimate children’s and parents’ experience and reduce suffering. By emphasising parenting factors in ADHD, they confirmed parents’ fears of being blamed and alienated them.	Participants may have been better informed about ADHD & more interested in it than the average British GP
Shaw et al. (2003) [43] Australia - Queensland	GP views: ADHD causes, role in diagnosis & management, behaviour therapy & medication Qualitative, as part of mixed methods study (see Shaw et al. 2002 [42])	28 GPs in 6 focus groups Random selection from RACGP Directory, (97 % response)	ADHD aetiology: Ineffective parenting, parent stress. Medicalisation of misbehaviour. Identified medical management (not parenting programmes, other family interventions). Little interest in management: time constraints, knowledge, training needs. Need diagnostic tool. Concern re media reports of diversion. Little guidance for GPs to determine symptoms or clinically significant impairment; research, guidelines do not encompass reality of GP clinical interview.	Random selection of GPs and excellent response allows high confidence in representativeness of findings
Salt et al. (2005) [40] UK – London	GP perceptions of ADHD and its management in primary care Mixed methods: Quantitative survey (see above) and focus groups	13 GPs (Focus groups) *Focus groups*: GP ADHD knowledge, beliefs (incl. aetiology, treatment); ability to recognise, diagnose; practice re referral, training, management	*Focus groups* GPs unsure of ADHD causes; controversial; new diagnosis; many lack confidence. Most refer, but not clear what they should report. Most recommended combination of meds and behaviour; several said no side effects or could not remember. None had ADHD training in basic medical education.	Convenience sample of GPs from one locality only; however can be linked to quan study (see above)
Dennis et al. (2008) [48] UK - London	Professionals’ and parents’ views of ADHD and service provision Qualitative	5 GPs; purposive sampling from GP practices; other health professionals recruited via professional networks. Purposive sampling of voluntary support groups for 49 parents. Focus groups, and semi-structured and narrative interviews in 2 London boroughs with 29 professionals in total (42 % response)	Professionals more likely to see ADHD as medical; parents more likely to ascribe to socio-environmental causes, often battled with professionals to see their viewpoint. Parent dissatisfied due to delayed diagnosis, inadequate information and lack of co-ordinated care. Professionals emphasised the need for multidisciplinary ADHD management. Non-compliance when parents had different views from professionals.	Small sample from each individual profession; purposive sample of GPs; limited reporting of findings for GPs

### Thematic narrative synthesis

Due to the heterogeneous and primarily descriptive nature of the research identified, a thematic narrative review and synthesis was carried out. For both quantitative and qualitative studies, responses were extracted and, in a line-by-line coding process, placed in conceptually related topic clusters or themes (Additional file 1: Table S1). The lower-level themes were then clustered into meaningful groups to arrive at a set of overarching themes from across the studies.

## Results

Eleven articles were identified in this review of GP attitudes in ‘gatekeeper’ settings where national ADHD guidelines require diagnosis in secondary, specialist services (Table 1).

One UK study examined predictors of GPs’ ADHD recognition and referral practice in a London community sample and a further 10 studies were descriptive: 6 surveys examining GPs’ self-rated ADHD attitudes and knowledge and 4 qualitative studies exploring GPs’ views in the UK and Australia. The studies were published between 2000 and 2010 with a bias towards earlier in the decade: six were of UK GPs [38–41], two from Australia [42, 43] and one each from Canada [44], Finland [45], and Iran [46]. Topics such as ADHD existence, aetiology, management, and GPs’ sources of information were common to several descriptive studies. As all questionnaires were study-specific and their content and response formats varied, each study is briefly summarised here (further details can be viewed in Table 1), after which the themes drawn from across them are presented.

### Community study of ADHD pathways

In a London community based study, Sayal and colleagues [38] examined predictors (GP and child/parent factors) of ADHD care pathways. Parents and teachers of children (5-11 years) registered with 10 London GP practices completed the Strengths and Difficulties Questionnaire (SDQ). In cases where GP recognised hyperactivity presentation, this always led to onward referral to specialist services or CAMHS. Factors that increased the likelihood of GP recognition, and subsequent referral, included parental recognition or referral request (OR 20.83, 95 % CI 3.05–142.08), and comorbid behaviour problems (OR 1.48, 95 % CI 1.04–2.12).

### Quantitative surveys of GP attitudes and knowledge regarding ADHD

Six studies surveyed GPs’ views and knowledge regarding aspects of ADHD, its nature, aetiology, diagnosis and treatment (Table 1). Ball [39] explored GPs’ views of ADHD diagnosis, treatment and training in South Wales, UK, nearly all of whom prescribed methylphenidate under specialist supervision. None believed a GP should diagnose, initiate prescribing, or monitor clinically. Most wanted clearer specialist advice and medication monitoring protocols and just 6 % had received formal ADHD training.

Shaw et al. [42] surveyed 399 GPs in Queensland, Australia on ADHD views and knowledge. Over half thought ADHD was over-diagnosed and three-quarters believed that children presenting with behaviour problems do not have ADHD. Nearly all stated family and social disruptions cause ADHD and three-quarters cited innate temperament or organic brain abnormalities. When asked to identify ADHD criteria from 16 DSM-IV symptoms for 5 conditions, over half were wrong on 8 or more symptoms. GPs engaged in some assessment activities but most would not be happy managing ADHD, offering time constraints and lack of knowledge as reasons. Nearly half considered stimulants the most appropriate first-line treatment and half cited behaviour therapy. However nearly one in five believed stimulants were always inappropriate and almost all agreed they have potential for abuse. Urban and female GPs were less likely to diagnose ADHD.

In Finland, Heikkinen and colleagues [45] measured primary care doctors’ self-evaluations of child psychiatry skills, including one question on ADHD. Asked if they felt well prepared to identify a child with various mental health difficulties, fewer doctors were confident for ADHD compared to either conduct disorder or depression, with almost half viewing themselves as poorly skilled in identifying a child with ADHD (40.7 %). Female GPs felt significantly more able to identify ADHD than male GPs but there were no gender differences for conduct disorder or depression.

Salt et al. [40] surveyed views and knowledge of UK GPs. Only a quarter believed ADHD to be well-defined. Almost all believed ADHD to be controversial, and believed a diagnosis could be stigmatising and disadvantageous. Most believed the media affect views of ADHD. Nearly half believed parents sought diagnosis to shift blame. Between a third and a half cited the following causes as ‘influential’ or ‘very influential’: genetic inheritance; chemical imbalance; brain damage or abnormality; other mental health disorders; and childhood psychological trauma. Similar proportions cited parenting quality. There was high accuracy in identifying key ADHD symptoms but most also (incorrectly) identified antisocial behaviour and educational underachievement as ADHD symptoms. Just 1 % were involved in diagnosis; three-quarters provided repeat prescriptions, mostly under specialist supervision. Most considered methylphenidate effective but one in five believed ADHD should not be treated with drugs.

Miller et al. [44] explored GPs’ self-perceived comfort and skill regarding child mental health and behavioural difficulties, including ADHD, in GPs in British Columbia, Canada. Nearly twice as many GPs cited low comfort and skill for diagnosing ADHD as did for diagnosing mood disorders. Similarly, two-thirds reported high comfort and skill for managing childhood mood disorders, yet only half did so for ADHD. Self-rated comfort and skill for ADHD diagnosis or treatment were positively associated with certification as a specialist in Family Medicine, participation in continuing medical education, and seeing more than five children a month in practice, and were negatively related to GP belief that ADHD is related to difficult stresses in the family, and that ADHD evaluation is often subjective and difficult.

In Iran, Ghanizadeh and Zarei surveyed GPs in Shiraz [46] about their ADHD-related views, knowledge and practices. One in ten had passed a special ADHD course; just under half believed they had adequate ADHD knowledge and relied on medical journals, media/ magazines and colleagues; and three-quarters believed ADHD should be managed by a psychiatrist. In terms of aetiology, nearly all believed poor parenting caused ADHD; half cited chaotic families and biological and genetic factors, and a third agreed that ADHD can often be caused by sugar or additives. Fewer than 1 in 10 agreed that ADHD-related difficulties are lifelong, and 1 in 5 believed ADHD was not serious and did not require management.

### Qualitative studies of GP attitudes and knowledge regarding ADHD

In five articles, GPs’ views were explored in more depth. Interviews with 10 London GPs were reported in Klasen [41] and Klasen and Goodman [47]. Three stances were found: (1) strong belief that labelling hyperactivity was not useful; (2) sceptical and discouraging medicalisation; and (3) believing that diagnosis was useful, providing access to school supports and a conceptual framework for parents (but only among GPs who had seen hyperactivity in the children of family or friends). Although several had academic appointments or a special interest in children’s services, GPs expressed scepticism about ADHD and confusion about variations in expert opinion. Most discouraged medicalisation and labelling, feeling labelling did more harm than good, perceiving it to be stigmatising and disempowering for a child, and self-fulfilling prophecy, leading to increased conflict between parent and child. They felt ADHD diagnosis was difficult and lacked the certainty they expect in practice. Most GPs believed hyperactivity was primarily due to poor parenting, ineffective discipline, or family stressors and that parents wish to medicalise it to avoid addressing this. They also stated their knowledge about hyperactivity was rudimentary; one reflected that ‘You have to learn all about these diseases that have a prevalence of about one in a million, and this relatively common problem is hardly mentioned’.

Shaw et al. [43] examined GPs’ views in Queensland, Australia. GPs believed ADHD was over-diagnosed, and often misdiagnosed where family/parenting problems or parent drug abuse were more relevant. They cited increased stressors and technology as promoting ADHD and were alarmed by newspaper articles about medication diversion. They believed GPs misdiagnose ADHD because of its complexity, and wanted a screening tool. Most believed ADHD was overmedicated and that family and parenting approaches should be applied more. Factors inhibiting GPs’ involvement in ADHD management were lack of knowledge and training; diagnostic complexity; the need for multi-disciplinary team and specialist involvement; and, importantly, time constraints in GP practices.

Salt et al. [40] explored 13 GPs’ views of ADHD in London with focus groups. All were unsure as to ADHD causes and highlighted its controversial nature, focusing on family dysfunction. Some believed ADHD was under-diagnosed compared to the US; others cited over-diagnosis. One described ADHD as fashionable ‘like dyslexia and all the rest’. More than half lacked confidence in recognising ADHD, stressing the need for specialist involvement; most said they would refer, but were not clear what they should report. All were aware of methylphenidate and most stressed a combined treatment approach. They were reluctant to engage in shared care and none had received undergraduate training a ‘new diagnosis’.

Finally, Dennis et al. [48] interviewed health professionals including five GPs in two London boroughs (districts). GPs talked about acceding to parental referral requests, especially in ‘risk’ situations and described some parents as expecting a ‘quick fix’. They had little interest in being more involved in ADHD care; most felt they could monitor physically or offer repeat prescriptions, but specialists should provide diagnosis and clinical management. Perceived barriers to GPs’ involvement were: a lack of ADHD training, complex prescribing, and time and resource constraints of general practice.

### Thematic narrative synthesis of GPs’ views and knowledge regarding ADHD

Themes and sub-themes were formed to reflect the findings from across the studies that addressed GPs’ beliefs regarding ADHD aetiology, diagnosis, treatment, training, and sources of information. Additional file 1: Table S1, displays the line-by-line analysis of all themes and sub-themes and gives an overview of locations and times for which these views were reported.

Themes were *Recognition rate*; *ADHD controversy (medicalisation, stigma, labelling); Causes of ADHD*; *GPs and ADHD diagnosis*; *GPs and ADHD treatment; GP ADHD training and sources of information; and Age, sex differences in knowledge and attitudes*.

#### Recognition rate

An ADHD screening study in the UK and GP self-reports in Australia and Canada suggested that GPs under-recognition of ADHD. In the UK (2002) only 1 in 10 children who screened positive for pervasive hyperactivity using the SDQ in a community sample were in contact with mental health services, even though 74 % of screened children had seen the GP in the previous year and GP hyperactivity recognition inevitably led to referral. In Australia (2002), GPs saw over 550 children aged 4–16 annually yet reported identifying just 1–5 ADHD cases in any given year, suggesting a recognition rate of under 1 %. In Canada (2005), 20 % GPs said they saw no children in whom they would consider ADHD; this compared to only 6 % who stated they saw no children in whom they would consider mood disorders, suggesting GPs recognised ADHD less frequently than other mental health difficulties in children, even though prevalence data indicate ADHD is more common.

#### ADHD controversy (medicalisation, stigma, labelling)

In surveys and focus groups in the UK, Australia and Iran (2000–2010), GPs typically expressed mixed feelings and scepticism about ADHD and about medicalising childhood behaviour, as well as concern about stigma. In the most recent study (Iran, 2010), 1 in 5 GPs believed ADHD was ‘not a serious problem’ that did not need to be managed and over 8 out of 10 GPs believed children with ADHD ‘misbehave primarily because they don’t want to obey rules and do their assignments. In Australia and the UK, GPs cited ADHD over-diagnosis and discussed overmedication, medicalisation of misbehaviour and suspicion about medicating children. GPs in London (2000, 2005, 2008) particularly felt that ADHD was controversial, potentially stigmatising and disadvantageous; it was considered a ‘fashionable’ diagnosis (2005), and referrals were characterised as a risk avoidance strategy (2008). Interviews in the UK (2000) identified three stances: strong anti-labelling; sceptical and reluctant to medicalise; or ADHD diagnosis viewed as facilitating parental understanding and school supports, but not something GPs wished to deal with. The UK studies also illuminated the concept of parent blame (see Additional file 1: Table S1), as GPs described parents as wanting to ‘shift blame’ or get a ‘quick fix’ for a child’s behaviour. Finally, in the UK, Australia and Iran, the media were described as influencing public attitudes to ADHD – including GP attitudes.

##### Causes of ADHD

GPs generally believed that ADHD was multi-factorial (Additional file 1: Table S1). Across times and locations, half to three-quarters of GPs (45–77 %) cited biological, neurological or related factors, but there was an equal or stronger emphasis on the influence of parenting, as half to nearly all (45–97 %) cited ineffective discipline, chaotic families, marital or family discord, or parental drug abuse. Nutrition was cited as a cause of ADHD by 12–37 % GPs in various settings and other environmental factors (school, media, and modern society) were cited by up to 25 %. Other factors were cited less frequently.

##### GPs and ADHD diagnosis

As would be expected in settings where guidelines do not recommend GP involvement in formal diagnosis, almost no GPs, just 1–5 %, in the UK, Iran & Canada, stated this was a suitable role for them (Additional file 1: Table S1). Of concern, however, was that almost all had low confidence in their recognition/diagnostic ability, citing their lack of training, and complexity and uncertainty regarding ADHD. Some also cited a need for good quality screening tools and clarity about what they should report when referring. Two studies (Finland 2002, Canada 2005), that asked GPs to self-rate ability to diagnose mental health difficulties including ADHD, found it was substantially lower for ADHD than for mood disorders in children (Additional file 1: Table S1). Two further studies (Australia 2002, UK 2005) assessed GPs’ ability to recognise ADHD symptoms: GPs’ identification of the three key ADHD symptoms of inattention, hyperactivity and impulsivity was very high, but many GPs mislabelled conduct and oppositional defiant symptoms as ADHD. This raises the possibility that when children present with ADHD symptoms alone, GPs may not recognise them as having ADHD.

##### GPs and ADHD treatment

As would be expected in gatekeeper settings, across studies, 71–94 % of GPs said the overview of a specialist was required to treat ADHD; ADHD symptom monitoring was not considered part of their remit, although half or more were prepared to engage in on-going prescribing and/or physical monitoring. Almost all who were asked had little interest in becoming more involved in ADHD care, citing lack of time and knowledge.

GPs expressed mixed views about treating children with medication generally. In Australia (2002), 43 % GPs felt stimulants were suitable first-line treatments, but 17 % said they were always inappropriate; in Iran (2010), 75 % agreed ADHD can be managed with medication, but 52 % of GPs believed only severe cases should receive it. In the UK (2005), most GPs stressed a combination approach to treatment (medication with psychosocial techniques).

##### GP ADHD training and sources of information

Most studies did not report on ADHD-specific GP training. Of those that did, just 6 % GPs in the UK and 10 % in Iran had received this. GPs in all qualitative studies frequently cited their lack of ADHD training and knowledge. Qualitative studies in Australia (2003) and the UK (2000, 2005) indicated that GPs relied on media as a source of ADHD information, as did about a quarter of GPs in surveys (UK 2001; Iran, 2010). All studies concluded that GP training in ADHD was needed.

##### Age, sex differences in knowledge, attitudes

Finally, just a few studies reported analyses of attitudes and knowledge by GP age or gender and there was no pattern of findings. Male GPs reported significantly higher comfort and skill in Canada, whereas female GPs did so in Finland (Additional file 1: Table S1). Age-related differences in GP knowledge were examined in just one Australia study but no significant differences were found.

## Discussion

This review aimed to identify GP attitudes and knowledge regarding ADHD in children, and their role in pathways to care, in jurisdictions where GPs are gatekeepers and where guidelines recommend diagnosis and treatment by specialist secondary services. This gatekeeper model is found in many countries in Europe, Canada, Australia, and elsewhere. The focus on such settings was chosen, as nationally representative or community-based studies in countries with gatekeeper models of service (e.g., Ireland, UK, the Netherlands) have found that only small proportions of children with ADHD receive treatment [6–8]. As GPs control access to ADHD diagnosis and services in such settings, their awareness of ADHD and attitudes to it can affect a critical junction in the pathway to care.

The systematic review of papers found surprisingly few studies of this topic, 11 studies (12 papers). Although diverse in focus, settings, time and measures, the studies were found to have explored common topics of *Recognition rate; ADHD controversy (medicalisation, stigma, labelling); Causes of ADHD; GPs and ADHD diagnosis; GPs and ADHD treatment; GP ADHD training and sources of information; and Age, sex differences in knowledge and attitudes*.

Strikingly, across this diverse set of studies, GPs’ views regarding causes, treatment and their role in ADHD were found to have many similarities and to reflect recent studies of other professionals, such as teachers, that highlight persistent of misconceptions about ADHD and its management [49]. As GPs are trained in the medical model, one might predict their interpretations of children’s ADHD-related difficulties would reflect the medical consensus, which is primarily biomedical. Such views, whilst present, were often overlaid with a (sometimes more prevalent) focus on the impact of parenting. Given the paucity and variability of the studies, the degree of GPs’ scepticism regarding the construct of ADHD, and of their negative views regarding paediatric ADHD medication, cannot be directly assessed, yet these beliefs or attitudes appeared across many settings and throughout the decade, reflecting other, more current reports of voices against medicalisation of ADHD [10, 17, 36]. Such perspectives occur also in teachers and society in general but are also reported (perhaps more alarmingly) in child and adolescent mental health services (CAMHS) where medication may be interpreted as a form of behaviour control and where competing ideologies regarding ADHD have been found to lead to inter-professional tensions [50]. Indeed, psychiatrists who support medication use and the existing ADHD evidence base also seek to bring the focus more to social, family and educational factors, asking about recent changes in the school system or society that may cause parents and teachers to feel less able to handle children with ADHD, and how they might be better supported [25]. In the US, rises in ADHD diagnoses in specific districts, line with the introduction of mandatory testing in schools suggests that the nature of the school environment, and/or performance pressures on schools and districts, may underlie rising ADHD diagnosis rates [10]. In sum, the variety of GPs’ views found in the present study, and their concerns regarding medicalisation, are seen also in views of other professionals, including those in mental health [50, 51], and continue to contribute to the socially contested nature of ADHD [1, 2, 10, 17, 19].

The presence of such views raises concern about GPs’ role in recognising ADHD in countries where they control children’s access to services. This is supported by UK research of the pathway to ADHD care, which indicates that children with hyperactivity, but without additional behaviour problems, are likely to go unrecognised by the GP unless the parent requests referral [38]. It is further supported by studies of parents, who have stated that GP scepticism regarding ADHD, and focus on parenting advice, has prevented discussion of children’s difficulties [47].

The finding that GPs are reluctant to be involved in active ADHD care also raises questions about policy recommendations for shared care between mental health services and primary care such as the UK NICE Recommendations [26]. For example, recent reports from the UK identified poor uptake in shared-care prescribing for ADHD and GPs’ concerns for the robustness of the diagnosis [52]. In Ireland, nearly two-thirds of GPs foresaw difficulties with potential shared care between primary care and psychiatric services [53] and only one-third to half of GPs are reported to have had any formal training in, or experience of working with, mental health issues in general [54]; the proportion with ADHD training is likely to be lower [55]. This raises concerns about the level of oversight being delivered to the substantial proportion of young people with ADHD who are transferred to GP care on leaving child and adolescent mental health services where adult services typically do not take on such cases [55]. Alternatives have been proposed, such as a General Practitioner with Special Interest (GPwSI) specialist model in the UK [56]. In the Netherlands, a recently published controlled study protocol for children with ADHD aims to reduce time between referral and start of treatment, with accelerated diagnosis and treatment plans, an online elearning training for primary care physicians, and enhanced co-operation between primary and specialist care in child mental health [57].

### Strengths and limitations

This review benefited from a systematic, wide-ranging search and is the first examination, to our knowledge, of international GP/FP attitudes to ADHD in jurisdictions where GPs are gatekeepers to ADHD services. It captures views across four continents in the first decade of the 21^st^ century. Several studies were of large, randomly selected samples with very good response rates (52–76 %) and a combination of quantitative and qualitative studies enables both depth and breadth to be accessed. At the same time however it should be noted that only a limited number of studies was identified and some were opportunistic surveys. The use of different questionnaires in each study means that caution should be employed when drawing conclusions about attitudes across these studies, and direct comparisons of proportions of GPs holding certain views across settings and times are not possible.

Furthermore, a skew towards the earlier part of the decade means that many studies report attitudes that are over a decade old. The limited range of studies (and paucity of recent findings) is surprising and suggests an urgent need for updated and more wide-ranging research in this field: it is possible that GPs’ views may have changed and may be influenced by the growing literature supporting biological contributions to ADHD [17, 21]. One study of primary school teachers’ attitudes to ADHD in Ireland [58] reported knowledge to be greater relative to previous studies. However as this was a small local study its findings remain to be supported in Ireland and elsewhere. A recent review on ADHD perceptions among healthcare professionals other than GPs as well as teachers and the general public found persisting misconceptions regarding ADHD etiology and treatment in settings ranging from Sri Lanka to Canada [46]. The authors conclude that these misconceptions reinforce ADHD stigma, and called for continued education about ADHD for all relevant professionals [46].

Indeed the possible role of stigma in GPs’ ADHD recognition and referral process is another aspect of ADHD that is worthy of further exploration. Stigma is substantially under-investigated regarding ADHD, [57] surprisingly so given Goffmann’s (1963) prediction that stigma is more likely to be triggered for conditions whose origins are uncertain or whose symptoms are thought to be under the individual’s control [59] – features of some GPs’ views of ADHD identified in this review. Some GPs were reported to be reluctant referrers, wishing to prevent children being exposed to the stigma of a diagnosis, and expressing a fear of ‘labelling’ that has been noted by others [13]. Although this stance regarding the adverse effect of a diagnosis may be held with protective intent, it may ultimately be to the detriment of a child with ADHD should it preclude access to treatment.

### Implications for training and services

GP education is one strategy to improve knowledge and self-efficacy regarding mental health in general in children and adults, and ADHD in particular, and can assist with appropriate referral to services [60–62]. As no definitive interventions or feasibility studies aiming to enhance identification of ADHD in primary care were identified in this review, this is an evident avenue that requires further exploration.

However, it should be noted that training and increased knowledge alone may not be enough to affect GPs’ behaviour, [62, 63]. Sikorski and colleagues, in a 2012 systematic review of randomised control trials of GP training in the care of depression, found very few studies; the existing evidence led to the conclusion that GP training alone does not improve care, but that training effects were more promising when combined with implementation of guidelines, and that GP training needs to be linked with more collaborative care models.

Notably, Canadian GPs, who had received autism education, only raised a concern with parents regarding possible symptoms of a child’s autism if linked with personal *certainty* about their clinical findings (supported by knowledge, screening and checklist tools) but also a sense of *urgency* about taking this action [62]. This finding, if it is generalisable to ADHD, may be particularly pertinent, as GPs not only lack training but may also be sceptical and hence may lack a sense of ‘urgency’ regarding referral and treatment. Furthermore, to address the question of urgency, research suggests that it would be necessary to train teachers, as well as to educate parents and other relevant family members. This is because studies of pathways to care [6, 13] and major reviews [64] indicate that service access is affected by parents’ and teachers’ knowledge about ADHD and their interpretation of child behaviour, as well as referral requests by parents. An exploration of such approaches would seem warranted for ADHD, i.e., combining training, guideline implementation, checklists, and collaborative care, with wider education for relevant professionals and society.

In relation to services, in settings where national or professional ADHD guidelines require secondary services to diagnose and initiate treatment, they often also (as, e.g., the UK NICE ADHD Guidelines) recommend the involvement of GPs in shared care models [26]. This was reiterated most recently in 2015 by Sayal [65] who noted the need for involvement of primary care service partners, given the extent of ADHD prevalence and constraints on secondary mental health service budgets. As this study indicates mixed attitudes to the diagnosis itself among GP and, crucially, very little interest at all in shared care, the importance of identifying GPs’ *current* ADHD attitudes and knowledge is highlighted once again.

## Conclusions

This review identifies knowledge gaps among GPs regarding ADHD in many jurisdictions where they operate as gatekeepers to ADHD care. Despite the limited number of studies found, their geographic breadth indicates that mixed (and often unhelpful) attitudes regarding the construct of ADHD are internationally widespread, even among medically trained clinicians, in these gatekeeper settings. Combined with prevalence studies and investigations of pathways to care, the findings suggests that education about ADHD is required not only for GPs but for all groups in the care pathway, including parents, teachers and the public, to enable parental concerns to be correctly identified and managed, and to ensure appropriate access to specialist services. This training might address barriers to parents’ perceptions of problems and to expression of concerns regarding possible ADHD in primary care consultations. It would also be valuable to work with GPs, to ensure that they are alert to relevant concerns and symptoms, and have adequate training and supports to respond accordingly.

## Additional file

Additional file 1: Table S1. Themes developed from review of gatekeeper GP attitudes and knowledge regarding ADHD. (PDF 1 mb)

## Abbreviations

ADHD: Attention-deficit hyperactivity disorder
CAMHS: Child and adolescent mental health services
DSM-IV: Diagnostic and Statistics Manual IV of the American Psychiatric Association
FP: Family practitioner
GP: General practitioner
UK: United Kingdom of Great Britain and Northern Ireland
US: United States of America
