# Supplementary material for: What do general practitioners know about ADHD? Attitudes and knowledge among first-contact gatekeepers: systematic narrative review
Source: BMC Fam Pract. 2016 Sep 7;17(1):129. doi: 10.1186/s12875-016-0516-x (PMC5013633; doi:10.1186/s12875-016-0516-x)
Supplement: Additional file 1: Table S1. — Themes developed from review of gatekeeper GP attitudes and knowledge regarding ADHD. (PDF 1 mb) [file 12875_2016_516_MOESM1_ESM.pdf]

Additional file 1: Table S1. Themes developed from review of gatekeeper GP attitudes and knowledge regarding ADHD

Recognition rate	
GP non-recognition was main barrier to specialist services. Only 12% children with pervasive hyperactivity in a community sample were in contact with CAMHS, though 74% had seen GP in past year. Only comorbid conduct problem or parent referral request predicted GP recognition.	[UK, 2002]
GPs diagnosed only 1–5 ADHD cases a year, yet saw >550 children 4–16 annually, so under-diagnosis likely.	[Australia, 2002]
20% of GP/FPs said they saw *no* children in whom they would consider or diagnose ADHD and only 6% did so for other conditions	[Canada, 2005]
ADHD Controversy (medicalisation, stigma, labelling)	
Overdiagnosis, misdiagnosis, overmedication	
ADHD over-diagnosed 55%	[Australia, 2002]
Children with behaviour problems do not have ADHD 76%	[Australia, 2002]
Children with ADHD misbehave because they don't want to obey rules, do assignments 83%	[Iran, 2010]
ADHD is not serious 20%	[Iran, 2010]
Many GPs did not know the level of ADHD diagnosis and thought it was both over- and underdiagnosed	[UK, 2005, FG]
ADHD is over-diagnosed; is misdiagnosed instead of parenting problems; is overmedicated; family and parenting approaches should be applied more	[Australia, 2003, FG]
GPs had reservations about methylphenidate - suspicious about use of drugs in children & long-term developmental effects	[UK, 2005, FG]
Controversial diagnosis, medicalisation	
ADHD - Medicalisation of misbehaviour	[Australia, 2003, FG]
Controversial 79%	[UK, 2005]
ADHD was well-defined 28%; It is hard to know when behaviours are age-related or due to ADHD 42% i	[UK, 2005]
Controversial; ‘new’ diagnosis; ADHD described as fashionable ‘like dyslexia and all the rest’	[UK, 2005, FG]
Divided opinions surrounding the disorder, and difficulty in diagnosis	[UK, 2005, FG]
GPs were: sceptical, discouraging of medicalisation and labelling predominant; aimed to demedicalise hyperactivity and reframe it as behavioural	[UK, 2000, Interviews]
Decisions about referral were moral as well as medical	[UK, 2000, Interviews]
Stigma	
Children can be stigmatized, disadvantaged by ADHD diagnosis 79%	[UK, 2005]
diagnosis can stigmatise, and make children passive and dependent	[UK, 2000, Interviews]
most felt labelling did more harm than good, fearing it could stigmatise a child, create a self-fulfilling prophecy, disempower the child, or	[UK, 2000, Interviews]

increase conflict between parent and child<br>reticent about making diagnosis because of negative consequences of labelling and treatment contentious	[UK, 2000, Interviews]
Parent blame	
Nearly half of GPs (44%) believed that parents were invested in child ADHD diagnosis as it shifts blame	[UK, 2005]
Parents expect a 'quick fix'	[UK, 2008, Interviews]
Most believed ADHD was primarily due to poor parenting, ineffective discipline, inability to set boundaries, or internal, social and environmental family stressors; and believed parents wished to medicalise hyperactivity, or exaggerated behavioural difficulties; did not recognize diagnosis may legitimate children's and parents' experience; and confirmed parents' fears of blame. Their beliefs clustered into 3 groups (most in 1 or 2) (1) ADHD labels and disempowers active children; reframed ADHD as poor parenting; (2) Sceptical, confused by contradictory expert opinions; discouraged medicalization, diagnosis was stigmatising; (3) Sceptical, diagnosis can be useful; aware of own limitations; sympathetic attitude to parents	[UK, 2000, Interviews]
Media influence attitudes	
Media influences attitudes to ADHD 90%	[UK, 2005]
GPs alarmed by newspaper articles about medication diversion	[Australia, 2003, FG]
25% get their ADHD knowledge from the media	[Iran, 2010]
21% get their ADHD knowledge from the media	[UK, 2001]
Causes of ADHD	
All GPs in focus groups were either unsure of the cause of ADHD or stated it was still unknown	[UK, 2005, FG]
Family, parenting factors	
Family disruption 97%; parenting 77%; poor discipline 75%	[Australia, 2002]
quality of parenting 50%, family type 45%;	[UK, 2005]
chaotic, dysfunctional family, 53%; parenting, spoiling, 90%	[Iran, 2010]
Ineffective parenting, parent stress	[Australia, 2003, FG]
marital or family discord and dysfunction	[UK, 2005, FG]
Child temperament, choices as factors	
temperament 77%	[Australia, 2002]
Misbehave primarily because they don't want to obey rules and do their assignments 82%	[Iran, 2010]
Genetic, biological, or neurological factors	
brain abnormality 70%	[Australia, 2002]
genetics 60%	[UK, 2005]
chemical imbalance 50%	[UK, 2005]
brain damage/abnormality 40%	[UK, 2005]

biological/genetic 47%	[Iran, 2010]
Early trauma	
childhood psychological trauma 40%<br>birth trauma 4%	[UK, 2005]<br>[Australia, 2002]
Nutrition	
food 12%<br>nutrition 25%<br>37% sugar, food additives	[Australia, 2002]<br>[UK, 2005]<br>[Iran, 2010]
Technology	
TV 7%; video games 5%<br>increased stressors and technology	[Australia, 2002]<br>[Australia, 2003, FG]
Education	
education 3%<br>school/education 30%	[Australia, 2002]<br>[UK, 2005]
Other	
social class 10% ethnicity 5% poverty 15%<br>peers 15%<br>environment chemicals 10%	[UK, 2005]<br>[UK, 2005]<br>[UK, 2005]
Prognosis	
ADHD is lifelong 7%<br>ADHD exists after childhood 85%	[Iran, 2010]<br>[UK, 2005]
GPs and ADHD diagnosis	
GPs' rate of involvement in diagnosis and their view of the role of specialists	
1% involved in formal diagnosis	[UK, 2005]
5% assess and manage children with probable ADHD themselves, without referring to experts	[Iran, 2010]
5% would evaluate and manage ADHD themselves. All others would refer out – for consultation (26%), for management (27%), for evaluation and management (19%) or for evaluation only (21%)	[Canada, 2005]
For children with ADHD, significantly more GP/FPs referred than handled themselves (no the case for social-emotional difficulties or mood disorders)	[Canada, 2005]
No GP thought GPs should initiate prescribing	[UK, 2001]
GPs identified their role as provisional diagnosis, referral; Most felt assessing children best undertaken by specialists within multi-disciplinary teams	[Australia, 2002]

Most refer… stressed importance of specialist involvement in diagnosis	[UK, 2005, FG]
No GP had ever given a diagnosis; believed to be task of specialists	[UK, 2000, Interviews]
Self-rated confidence in diagnosing ADHD	
GPs reticent about making ADHD diagnosis; because of inadequate training, many lack confidence	[UK, 2005, FG]
Over half lacked confidence in recognizing the condition. Not clear what they should report when referring	[UK, 2005, FG]
40% GPs felt unprepared to identify a child with ADHD (compared to 20% for conduct disorder, 30% for depression)	[Finland, 2002]
For diagnosing ADHD, family physicians rated their comfort/skill as substantially lower than for diagnosing mood disorders (48% reported low comfort, 52% low skill for ADHD compared to 27% low comfort, 31% low skill for mood disorders).	[Canada, 2005]
Comfort/skill relating to ADHD - Positively related to having participated in CME; See more than 5 children per month with ADHD; Certified as specialist in Family Medicine	[Canada, 2005]
Comfort/skill relating to ADHD: Negatively related to Belief that these problems are related to stresses in the family that are hard to manage and Belief that evaluation of these problems is often subjective and difficult	[Canada, 2005]
GP/FPs' likelihood of referring rather than handling themself - The proportion of variance explained by Comfort/Skill was just 34% for ADHD, compared to 53-61% for behavior, social-emotional or mood disorders.	[Canada, 2005]
Self-reported comfort/skill an important predictor of GP/FP taking primary responsibility for a case, and self-reported comfort/skill was in turn related to previous educational exposure to this field, and beliefs about mental health problems in children	[Canada, 2005]
GP had weak knowledge of ADHD & comorbidities; low confidence diagnosing, managing	[Australia, 2002]
Lack of training and education regarding ADHD a barrier to more involvement	[UK, 2008, Interviews]
ADHD symptom and diagnostic knowledge	
When shown 16 DSM-IV symptoms for oppositional defiance disorder (ODD), conduct disorder, anxiety, depression, and ADHD and asked to identify ADHD criteria, 58% GPs were wrong for half or more ADHD symptoms. A quarter to a third identified ODD criteria as ADHD: blaming others; destroying property; initiating fights; spiteful or vindictive; physically cruel	[Australia, 2002]
Most GPs correctly included inattention, hyperactivity, impulsivity as diagnostic criteria, but 75% or more cited 'educational underachievement', 'antisocial behaviour' and 'sleep problems' as ADHD symptoms	[UK, 2005]
GPs were not sure about diagnostic procedures – one would observe the child in the consultation; none mentioned specific rating scales	[UK, 2005, FG]
GPs have little guidance to determine symptoms or clinically significant impairment. Research and guidelines do not encompass reality of GP clinical interview. Need diagnostic tool.	[Australia, 2003, FG]
GPs' lack of training and education regarding ADHD is a barrier to more involvement re diagnosis	[UK, 2008, Interviews]
GPs and ADHD treatment	
GP involvement in treatment	
94% GPs need overview of child psychiatrist or paediatrician to prescribe methylphenidate	[UK, 2001]
GPs overloaded, reluctant to take on more. 54% said primary care could monitor physically; 64% psychiatry should monitor clinically	[UK, 2001]

Specialist should be manage and monitor. 73% engaged in repeat prescribing, but less than half, 34%, monitored (14% alone, 20% with specialist)	[UK, 2005]
Most GPs would not be happy managing ADHD, due to time constraints (91%), lack of knowledge (74%)	[Australia, 2002]
ADHD should be managed by a psychiatrist 71%; only 18% prepared to manage in follow-up	[Iran, 2010]
GPs identified their roles as monitoring assistance (height, weight, appetite, sleep); psycho-education; school liaison.	[Australia, 2003, FG]
Low interest in being involved in ADHD care (compared to e.g., health visitors and school nurses); Other experts manage clinically	[UK, 2008, Interviews]
Barriers to involvement were time and resource constraints of general practice, complex prescribing, lack of training	[UK, 2008, Interviews]
Barriers to greater GP involvement: Resources; addiction concern; child behaviour problems complex; lack of ADHD training.	[Australia, 2002]
For managing ADHD, under half (49%) reported high comfort and skill compared to two-thirds (65%) for childhood mood disorders	[Canada, 2005]
Appropriate treatment	
Stimulant use with children always inappropriate 17%, stimulants can be abused 86%, they are addictive 40% [Australia, 2002]	
1st line treatment: Behavioural 51%, stimulants 43%	[Australia, 2002]
92% methylphenidate, followed by family therapy (74%) & behaviour therapy (64%). In focus groups, most recommended a combination	[UK, 2005]
ADHD can be managed with medication 75%; 52% against its use unless severe	[Iran, 2010]
GP ADHD training and sources of information	
Knowledge (see also ADHD symptom and diagnostic knowledge, above)	
Several said no side effects of medication, or could not remember.	[UK, 2005, FG]
Fewer than half of the GPs, 47%, believed they had adequate knowledge about ADHD	[Iran, 2010]
GPs said their knowledge about hyperactivity was rudimentary	[UK, 2000, Interviews]
Training and sources of ADHD information	
6% formal ADHD training [UK, 2001]	
10% passed special courses on ADHD [Iran, 2010]	
32% info from medical journals, 25% media, magazines, 18% from colleagues	[Iran, 2010]
29% journal article, 21% media e.g., television, magazines, 5% conference/ course	[UK, 2001]
Lack of ADHD training, education noted	
84% wanted further ADHD training (68% preferred tutorial or lecture, 27% written, 5% phone)	[UK, 2001]
Lack of training and education regarding ADHD	[UK, 2008, Interviews]
GPs noted a lack of knowledge and training	[Australia, 2003, FG]
GPs did not have adequate training in ADHD recognition. None had received ADHD training in basic medical education – a 'new diagnosis'	[UK, 2005, FG]
GP: 'You have to learn all about these diseases that have a prevalence of about one in a million, and this relatively common problem is hardly mentioned'	[UK, 2000, Interviews]
GPs wanted greater knowledge; lack of ADHD training a barrier to involvement	[Australia, 2002]

Age, sex differences in knowledge and attitudes	
Male GPs had sig more higher-rated comfort/skill re ADHD	[Canada, 2005]
Female GPs considered they were significantly more able to identify ADHD than male GPs did	[Finland, 2002]
Female GPs identified more ADHD criteria correctly than men; were less likely to diagnose ADHD compared with men; and were more likely to agree or strongly agree that video games and television watching contribute to ADHD development	[Australia, 2002]
GPs aged 41–50 years outperformed all other groups in correctly identifying ADHD criteria	[Australia, 2002]
Non- significant trends indicated more older GPs believed sole parenting or junk food contributed to ADHD and fewer believed declining education standards did so	[Australia, 2002]
Descriptives only reported - No analyses by age, gender, length of time in practice or any other factors	[Iran, 2010]**;** [UK, 2001]; [UK, 2005]
